# The psychology of romantic relationships: motivations and mate preferences

**DOI:** 10.3389/fpsyg.2023.1273607

**Published:** 2023-11-28

**Authors:** Eugene Tartakovsky

**Affiliations:** The School of Social Work, Tel Aviv University, Tel Aviv, Israel

**Keywords:** romantic relationships, romantic motivations, personal values, mate preferences, adolescents and young adults, Israel, Jews, Arabs

## Abstract

**Introduction:**

This study investigates motivations to engage in romantic relationships. We examine the structure of romantic motivations and their connections with personal values and mate preferences.

**Method:**

The study was conducted in Israel among young men and women looking for a romantic partner (*n* = 1,121, 40% male, age 18–30).

**Results:**

Data analysis demonstrated that basic romantic motivations form a circumplex that may be partitioned into four higher-order romantic motivations: love and care, family and children, status and resources, and sex and adventure. The romantic motivations formed a meaningful pattern of connections with higher-order values, thus confirming that context-specific motivations are derived from general motivational goals expressed in values. Personal value preferences and romantic motivations predicted the sought-after partner characteristics over and above sociodemographic variables. Values were indirectly (through romantic motivations) and directly connected to mate preferences.

**Discussion:**

The study advances our understanding of romantic relationships among young people and opens new directions for research and counseling.

*There are so many books on how to get married and not one on why. Anonymous*

## Introduction

This study focuses on the motivational aspects of romantic relationships. We define romantic relationships as those based on the emotional and physical attraction that could lead to long-term intimate relationships. We focus on young people looking for romantic relationships, i.e., those who presently have no romantic partner but are interested in finding a boy/girlfriend. We strive to understand what motivates young people to engage in romantic relationships, how their romantic motivations are related to the general motivational goals reflected in their value preferences, and whether romantic motivations and values can predict the sought-after characteristics of the partner.

Our study is based on the theory of human values (Schwartz et al., 2012; Schwartz, 2017). This general psychological theory presents a comprehensive system of human motivations corroborated as near-universal across different cultures (Schwartz et al., 2012; Sagiv and Schwartz, 2022). Numerous studies have demonstrated that values affect human cognition, emotions, and behavior (Schwartz, 2017; Sagiv and Roccas, 2021). However, they have rarely been applied to the study of romantic relationships.

### Motivations for romantic relationships

Previous studies on pre-marriage romantic motivations focused on motivations to engage in sexualized relationships – “hookups” (Uecker and Stokes, 2008; Townsend et al., 2020; Weitbrecht and Whitton, 2020; Thorpe and Kuperberg, 2021) and dating motivations (Rempel et al., 1985; Jones, 1993; Smiler, 2008; Keramat et al., 2013; Bryant and Sheldon, 2017; Timmermans and Alexopoulos, 2020). These studies assumed that people have numerous motivations to engage in romantic relationships. Thus, considering motivations for sexualized romantic relationships (hookups), researchers mention getting an experience, sexual experimentation, physical pleasure, fun, excitement, feeling attractive, escaping loneliness, increasing social status, answering social expectations, and following a social script (Uecker and Stokes, 2008; Weitbrecht and Whitton, 2020; Thorpe and Kuperberg, 2021). The list of dating motivations included social status, approval from others, new opportunities, sex, emotional support, adventure, curiosity, love, companionship, a step to marriage, care, and empathic concern (Rempel et al., 1985; Bryant and Sheldon, 2017). Several researchers clustered basic romantic motivations into higher-order motivations. One partition distinguished between autonomous (e.g., fun) and non-autonomous motivations (e.g., fulfilling others’ expectations) (Townsend et al., 2020). Another classification distinguished between extrinsic (e.g., social status), instrumental (e.g., emotional support), and intrinsic (e.g., mutual comfort) romantic motivations (Rempel et al., 1985).

Other researchers focused on marriage motivations. (Eekelaar, 2007; Park and Rosén, 2013; Czyżkowska and Cieciuch, 2020). Thus, in a qualitative study conducted among women in the UK, the participants reported that marriage provided them with reproductive, financial, and legal security (Carter, 2018). Specifically, they noted that marriage raised their social status, provided them with economic resources, and increased the security of their children. Moreover, many women connected marriage with tradition. They also said marriage is desirable because it is traditional, natural, and “normal”; not marrying is undesirable, abnormal, and socially unacceptable. Another qualitative study in the UK demonstrated that some people marry because they comply with the convention, i.e., follow religious rules or prescriptions, social or cultural practices, and their parents’ wishes (Eekelaar, 2007).

A quantitative study conducted in the US found six reasons for marriage: romance, respect, trust, finances, meaning, and physical (Park and Rosén, 2013). A 2010 Pew Research Center survey investigating the reasons to marry in the US found that love, indeed, wins all, followed by companionship, having children, and financial stability. Answering the question about the advantages of being married over single, respondents mentioned having a fulfilling sex life, being financially secure, finding happiness, getting ahead in a career, and having social status (Cohn, 2013).

Studies conducted in Russia distinguished between biological, sociocultural, economic, and psychological motives of marriage (Fedoseeva and Ivanova, 2018). Among the most common motives were an escape from parents, a sense of duty, an escape from loneliness, and following a tradition. Love, prestige, and the search for material wealth took the last places in this ranking. In addition, the following reasons were mentioned: understanding, psychological support, being an authentic self, self-realization, and having and raising children. Finally, a study conducted in Nigeria found that when considering marriage, people consider parental pressure and social norms, economic survival, connection with wealthy and powerful individuals, domestic help, guaranteed support, and reproductive tasks (James, 2010).

The literature review demonstrates that in most studies, romantic motivations were used as a list of non-related entities; they remained unsystematized (for exemptions, see Rempel et al., 1985; Jones, 1993; Townsend et al., 2020), and the connections between them remained unclear (Eekelaar, 2007; James, 2010; Cohn, 2013; Park and Rosén, 2013; Hurt, 2014; Carter, 2018; Fedoseeva and Ivanova, 2018; Thorpe and Kuperberg, 2021). Most existing studies on romantic motivations are not theory-driven. Therefore, we need a theory that will permit us to systematize numerous motivations for romantic relationships into a meaningful structure and explain connections between them and other variables.

### Theory of human values

Values are cognitive constructs defining desirable trans-situational goals and ordered by importance; they represent people’s motivations and provide a basis for attitudes and behavior (Schwartz, 2006). The present study is based on Schwartz’s theory of values (Schwartz et al., 2012; Schwartz, 2017). In its most recent formulation, the theory specifies a comprehensive set of 19 motivationally distinct values: power (dominance and resource), achievement, hedonism, stimulation, self-direction (thought and action), universalism (nature, concern, and tolerance), benevolence (caring and dependability), humility, tradition, conformity (rules and interpersonal), security (personal and social), and face (Schwartz et al., 2012).

The theory assumes the existence of dynamic relations between the values in that the pursuit of each value has consequences that may conflict or may be congruent with the pursuit of other values. The conflicts and congruities among basic values yield an integrated structure of four higher-order value types arrayed along two orthogonal dimensions: self-transcendence vs. self-enhancement and openness to change vs. conservation. Openness to change values (including self-direction and stimulation) emphasize readiness for new ideas, actions, and experiences. They contrast with conservation values (including conformity, tradition, and security) that emphasize self-restriction, order, and preserving the status quo. Self-enhancement values (including power and achievement) emphasize pursuing one’s interests. They contrast with self-transcendence values (including universalism and benevolence) that emphasize transcending one’s interests for the sake of others. Three values overlap between two higher-order value types: face (conservation and self-enhancement), hedonism (openness and self-enhancement), and humility (self-transcendence and conservation) (Schwartz et al., 2012).

Researchers assume that personal value preferences affect the individual’s attitudes, behavior, and emotions because values express general motivational goals in human life (Schwartz, 2017). Several psychological mechanisms explaining the effect of values have been suggested; however, the valence mechanism is probably the most crucial (Hitlin, 2003; Sagiv and Roccas, 2021). This mechanism assumes that people choose specific attitudes, behaviors, and emotions to attain the general motivational goals reflected in their value preferences (Schwartz, 2017). The existence of the valence mechanism has been confirmed in numerous studies regarding a wide range of behaviors and emotions (Tamir et al., 2016; Sagiv and Roccas, 2021); however, it has not been investigated in the context of romantic relationships.

### Conceptualization of romantic motivations

We assume that general motivational goals expressed in personal value preferences provide a foundation for all other motivations in human life. We further assume that people formulate (or may formulate) specific motivational goals they strive to achieve in each context. The context-specific goals are derived from general motivational goals reflected in values. The connection of context-specific to general goals is twofold. First, the content of each context-specific goal is related to the corresponding general motivational goal (or several such goals). Second, the structure of context-specific goals (the commonalities and contradictions between them) parallels (probably, with some exemptions) the values’ structure. That means that basic context-specific motivations constitute a circumplex that may be divided into higher-order motivations that parallel higher-order values. Finally, we assume that general motivational goals affect attitudes and behaviors directly and indirectly through their connections with context-specific motivations. The idea of context-specific motivations connected to values has been recently suggested for investigating copying with COVID-19 and the energy crisis (Liscio et al., 2022), artificial intelligence (Masso et al., 2023), and marriage (Czyżkowska and Cieciuch, 2020). In the present study, we apply the concept of context-specific motivations to the investigation of romantic motivations.

Developing the concept of romantic motivations, we assumed that when looking for a partner, people aspire to attain motivational goals that are attainable in romantic relationships. We further assumed that individuals derive their romantic motivational goals from their general motivational goals expressed in their value preferences. Therefore, romantic relationships are a vehicle for attaining specific motivational goals that express general motivational goals in the context of romantic relationships. Thus, romantic motivational goals may have different importance across individuals following their value preferences. Finally, we assumed that the choice of a romantic partner depends on the motivational goals of the individual, i.e., people look for a partner who will best help them attain their motivational goals.

### Building the romantic motivations scale

We built the scale measuring romantic motivations in several steps. First, we collected romantic motivations mentioned in the research literature and, when required, reformulated them to fit the situation of looking for a boy/girlfriend. In addition, we conducted interviews with about 80 young people from different ethno-religious groups in Israel, asking them about their motivations for seeking a girl/boyfriend. Thus, we created a comprehensive list of romantic motivations. After that, with a group of students applying the inter-judges’ agreement, we discarded repeated items and reformulated some items to make them clearer (Taherdoost, 2016). Then, we used the inter-judges’ agreements with a colleague researcher, an expert in value theory, to decide to which basic motivation each item belongs and to which higher-order motivational cluster each basic romantic motivation belongs, paralleling the values’ circumplex (Schwartz et al., 2012; Czyżkowska and Cieciuch, 2020). Thus, we formulated 14 basic motivations people pursued in their quest for romantic relationships and generated a list of items measuring each motivation. We did not find romantic motivations related to security (social), conformity (rules), humility, and universalism values. Motivational goals represented in these values are probably unattainable in romantic relationships. Table 1 lists values, general motivational goals, and basic romantic motivations. Appendix Table A1 presents basic romantic motivations and the corresponding scale items.

**Table 1:** Values, general motivational goals, and basic romantic motivations.

Values	General motivational goals	Basic romantic motivations
Self-direction	Freedom to cultivate one’s ideas and abilities and determine one’s actions	Psychological growth
		Independence from parents
Stimulation	Excitement, novelty, and change	Escape from loneliness
Hedonism	Pleasure and sensuous gratification	Sexual satisfaction
Achievement	Success according to social standards	Social advancement
Power	Exercising control over people and resources	Control over the other
		Economic benefits
Face	Maintaining one’s public image and avoiding humiliation	Respect
Security (Personal)	Safety in one’s immediate environment	Receiving emotional support
		Feeling loved
Tradition	Maintaining and preserving cultural, family, or religious traditions	Starting a family
		Childbearing and childrearing
Conformity (Rules)	Avoidance of upsetting or harming other people	Avoiding social pressure
Benevolence	Being a reliable and trustworthy member of the group devoted to the welfare of group members	Care for the other

Based on the similarities between romantic motivations and general motivational goals reflected in values, we hypothesized that basic romantic motivations form a circumplex paralleling the values’ circumplex, which might be partitioned into four higher-order romantic motivations (clusters of basic romantic motivations), each related to a higher-order value (H1):

1. A cluster related to openness to change values includes the following romantic motivations: psychological growth, independence from parents, escape from loneliness, and sexual satisfaction.
2. A cluster related to self-enhancement values includes the following romantic motivations: social advancement, control over the other, economic benefits, and gaining respect.
3. A cluster related to conservation values includes obligations to raise a family, finding a partner for childbearing and childrearing, resolving social pressure to find a partner, finding emotional support, and feeling loved.
4. Care for the other through romantic relationships is related to self-transcendence values.
5. Romantic motivations derived from opposing higher-order values are located on opposite sides of the circumplex: the first and third motivational clusters oppose each other, as well as the second and fourth clusters.

### The effect of socio-demographic variables on romantic motivations

Several studies have investigated the effects of socio-demographic variables on romantic motivations. When reporting on their motivation for hookups and other sexualized romantic relationships, women placed a greater emphasis on love, commitment, and initiating or solidifying relationships, while men were more likely to endorse pleasure, self-affirmation, status, and peer conformity as their motives (Smiler, 2008; Weitbrecht and Whitton, 2020; Thorpe and Kuperberg, 2021). The gender differences in marriage motivations indicated that women more than men marry for economic security and religious reasons, while men more often than women seek the satisfaction of sexual needs (Blakemore et al., 2005; Spivey, 2010; Gittins, 2017; Carter, 2018).

Data regarding ethnic and racial differences in romantic motivations is scarce. In one study, men and women of color in the US reported a stronger motivation for sex in hookups (Uecker et al., 2015). However, when considering marriage, black women reported a stronger economic motivation than white women, and both genders reported more religious motivations for marriage than whites (Bulcroft and Bulcroft, 1993; Edin, 2000; Hurt, 2014).

The motivation to create a family was stronger among young religious dating people than non-religious people (Fuller et al., 2015). At the same time, highly educated and non-religious young people reported stronger marriage motivations related to sex, pleasure, and mutual care (Eekelaar, 2007; Hurt, 2014; Fedoseeva and Ivanova, 2018).

Previous studies applied evolutionary and biosocial role theories to explain the effects of socio-demographic variables on romantic motivations (Bulcroft and Bulcroft, 1993; Eekelaar, 2007; Smiler, 2008). In the present study, we applied the values theory to formulate our hypotheses on the effect of socio-demographic variables on romantic motivations (Schwartz, 2017). As explained above, we assumed that romantic motivations are derived from general motivational goals expressed in personal value preferences. Therefore, we assumed that connections of socio-demographic variables with romantic motivations parallel their connections with values. The connections between socio-demographic variables and values are well-studied (Sagy et al., 2001; Schwartz et al., 2012; Schwartz, 2017; Walsh and Tartakovsky, 2021; Tartakovsky, 2023). At the level of higher-order values, a higher preference for openness to change and a lower preference for conservation values are associated with a younger age, being a male, a higher level of education, a lower level of religiosity, and being Jewish vs. Arab Israeli. A higher preference for self-transcendence and a lower preference for self-enhancement values is associated with an older age, being a female, being Jewish vs. Arab Israeli, and having a higher level of education. In line with the results of previous studies, we formulated the following hypotheses related to the connections between socio-demographic variables and romantic motivations:

*H2*: A higher preference for romantic motivations related to openness to change values and a lower preference for romantic motivations related to conservation values are associated with a younger age, being a male, a higher level of education, being a Jewish Israeli, and a lower level of religiosity.

*H3*: A higher preference for romantic motivations related to self-transcendence values and a lower preference for romantic motivations related to self-enhancement values are associated with an older age, being a female, being a Jewish Israeli, and a higher level of education.

### Mate preferences

Most existing studies on mate preferences have focused on the issues of universality in the ranking and gender similarities and differences (Buss et al., 2001). The results of these studies have been unequivocal: Both genders prefer a mate who is kind, intelligent, and healthy; however, there are cross-cultural gender differences related to the resources and fertility characteristics of the mate. Women, more than men, prefer long-term partners with the ability to acquire and confer resources, while men, more than women, prefer partners with high reproductive value, indicated by attractiveness and relative youth (Walter et al., 2020). Two theories explain the gender differences in mate preference. The evolutionary theory states that gender differences result from women facing a larger reproductive investment than men. Biosocial role theory claims that gender differences result from the behaviors that men and women cultivate based on societal expectations of gender roles (Buss et al., 2001; Thompson and O’Sullivan, 2012).

Another universal finding regarding mate preferences relates to assortative mating: In all cultures and social groups, individuals prefer partners similar to them (Thompson and O’Sullivan, 2012; Cooperman and Waller, 2022). Moreover, couples of similar spouses are more stable and happier in relationships (Buss et al., 2001; Luo, 2017). Different socio-psychological mechanisms explaining assortative mating have been suggested, including personal preferences, mating market operation, social homogamy, and convergence (Luo, 2017). No gender differences in assortative mating have been assumed, and we found no empirical studies on this issue. However, one study demonstrated that higher education was associated with a higher importance of similarity in the partner (Whyte and Torgler, 2017).

Few studies have focused on interpersonal differences in mate preferences and psychological theories explaining them. One such study applied attachment theory; however, it found that differences in attachment styles are not related to mate preferences (Cohen and Belsky, 2008). Another study investigated the connection between self-monitoring, dating motivations, and mate preferences (Jones, 1993). It found that high self-monitoring individuals (those who are attentive to the situation and interpersonal cues for appropriate behavior) preferred partners with high social status, sex appeal, and physical attractiveness, while low self-monitoring individuals (those who based their behavior on their attitudes, feelings, and beliefs) preferred partners high on honesty, loyalty, and similar believes and values. In addition, high self-monitoring individuals expressed more extrinsic motivations for dating, while low self-monitoring individuals expressed more intrinsic motivations for dating. Finally, one study applied Schwartz’s values theory (Goodwin and Tinker, 2002). This study has demonstrated that personal value preferences can explain individual differences in mate preferences. Specifically, the higher importance of conservation vs. openness to change values was associated with higher preferences for a partner who is a good earner, from a good family, healthy, a good housekeeper, and religious. The higher importance of self-enhancement vs. self-transcendence values was associated with preferences for a partner who is attractive, healthy, from a good family, wants children, is a good earner, a university graduate, and a good housekeeper. The present study investigates the connections between mate preferences and motivational goals, general (expressed in values) and context-specific (expressed in romantic motivations).

The present study focuses on three characteristics of the potential partner: socioeconomic status, physical attractiveness, and similarity. We chose these characteristics because they have been well-studied from the perspective of group differences/similarities, while the individual-level factors affecting them have rarely been investigated. In the present study, we assumed that individuals derive their mate preferences from their romantic motivations, i.e., they look for a partner to help them attain their romantic motivational goals. Individual mate preferences might also be connected to personal value preferences since the correctly chosen partner may help to attain one’s general motivational goals (Goodwin and Tinker, 2002).

We assume that romantic relationships with a high-status partner may permit individuals to raise their social status and achieve social dominance and control over resources by using the partner’s status and resources (Cohn, 2013; Gittins, 2017). Thus, seeking a high-status partner should be compatible with romantic motivations associated with self-enhancement values – dominance over others, control over resources, and demonstrating social success (Schwartz, 2017).

The partner’s physical attractiveness must be important for individuals seeking a romantic partner to satisfy sexual needs (Thompson and O’Sullivan, 2012; Gittins, 2017). In addition, people looking for social challenges and obtaining a new experience may also prefer a good-looking partner because it is more challenging to develop relationships with such a partner (Cohen and Belsky, 2008; Park and Rosén, 2013). Thus, the partners’ physical attractiveness may be compatible with romantic motivations associated with openness to change values.

Finally, having a partner similar to oneself is socially normative and promotes the preservation of the existing social order and tradition (Luo, 2017). It may also increase the individual’s sense of security (Schwartz C. R., 2013). Therefore, looking for a similar partner should be compatible with romantic motivations associated with conservation values (Schwartz, 2017; Czyżkowska and Cieciuch, 2020). Based on these assumptions, we formulated the following hypotheses related to connections between romantic motivations and mate preferences:

*H4*: The higher importance of social status in the romantic partner is connected to romantic motivations associated with self-enhancement values.

*H5*: The higher importance of physical attractiveness in a romantic partner is connected to romantic motivations associated with openness to change values.

*H6*: The higher importance of similarity in the romantic partner is connected to romantic motivations associated with conservation values.

*H7*: Romantic motivations partly mediate the connections between values and mate preferences, i.e., values are connected to mate preferences directly and indirectly through their connection to the corresponding romantic motivations.

## Methods

### Participants and procedures

This study used a community convenience sample of 1,121 participants (40% males). The mean age was 24.3 (*SD* = 3.11, range = 18–30). 79% of the participants had a tertiary degree or studied for such a degree. 70% of the participants were Jewish, 24% were Muslim, 5% were Christian, and 1% were Druze. 56% were secular, 29% were traditional (following some religious traditions and practices), and 16% were religious. Immigrants constituted 5% of the sample. Compared to the sociodemographic characteristics of young people reported by the Israeli Central Bureau of Statistics (2023), the following groups were slightly (10% or less) overrepresented in the sample: women, secular, highly educated, and Israeli-born. At the time of the study, all participants had no romantic partner; however, about 2/3 of them had such a partner in the past.

The Tel-Aviv University Review Board approved the study. Undergraduate students who participated in a senior research seminar (a third-year BA course) distributed the questionnaires as a part of the course requirements. Students participating in the seminar lived in different areas of the country, ensuring a geographically heterogeneous sample. Adults aged 18–30 who did not have a girl/boyfriend but would like to find one were invited to participate in the study. The anonymity of the participants was ensured, and all participants signed an informed consent form. The questionnaires were distributed using Google Forms through WhatsApp, Facebook, and e-mail. The participants did not receive compensation for completing the questionnaires. The study was conducted in Hebrew.

### Measures

#### Personal value preferences

Personal value preferences were measured using the Portrait Values Questionnaire, PVQ-R (Schwartz et al., 2012). This questionnaire consists of 57 items. Each item portrays an abstract person describing their goals, aspirations, and wishes that indicate the importance of a specific value. Respondents indicate how similar the described person is to them on a 6-point scale, from 1 (*not like me at all*) to 6 (*very much like me*). Item example (Conformity): “It is important to him/her to avoid upsetting other people.” Cronbach’s alphas of the four higher-order values were high: self-enhancement – 0.86, openness to change – 0.84, conservation – 0.86, and self-transcendence – 0.88. The higher-order values on the axes’ poles were strongly negatively correlated: *r* = −0.67 for openness to change – conservation and *r* = −0.61 for self-transcendence – self-enhancement. To avoid the multicollinearity problem, we used axes’ scores in all multivariate analyses, built by subtracting the scores of one pole of an axis from the other. This approach was suggested in several previous studies (Goodwin and Tinker, 2002; Abramson et al., 2018; Sverdlik and Rechter, 2020).

#### Romantic motivations

The scale measuring romantic motivations was created for the present study. The scale included 73 items allocated into 14 basic romantic motivations. Thus, each motivation was measured using 3–9 items. The participants were asked to what extent each motivation was important in their search for a girl/boyfriend. They answered on a 6-point scale, from 1 – *not important at all* to 6 – *very important*. Example items: “To feel loved.” “To avoid boredom.” “To have somebody who will buy me things.” “To satisfy my parents’ expectations.” The internal consistency of all 14 subscales measuring romantic motivations was high (Cronbach alphas 0.84–0.95). Appendix Table A1 presents romantic motivations and scale items with Cronbach alphas for each scale.

#### Mate preferences

We measured the importance of three characteristics of the potential partner: social status, physical attractiveness, and similarity. Mate preferences in status (4 items) and attractiveness (3 items) were measured using items from Buss et al. (2001). Items measuring similarity (5 items) were adopted from Buss et al. (2001), Schwartz (2013), and Luo (2017). The participants were asked how important it is to them that their girl/boyfriend would have specific characteristics. They answered on a 6-point scale, from 1 – *not important at all* to 6 – *very important*. Example items: “Has a high social status.” “Looks good.” “Has interests similar to yours.” To test for the structural validity of the scale, we conducted Exploratory Factor Analysis (EFA) separately for men and women. We used the Principal Component Extraction Method, Oblimin Rotation with Kaiser Normalization, and a fixed number of factors to extract. The results confirmed the scale’s structure. The total variance explained by the three-factor solution was 64% for men and 61% for women. As required, all first-factor loadings were higher than 0.40, with no second-factor loading higher than 0.30. Appendix Table A2 presents the EFA results. Internal consistency of the mate preference subscales measured by Cronbach’s α was high (men/women): 0.84/0.83 for status, 0.77/0.72 for physical attractiveness, and 0.80/0.76 for similarity.

As recommended in previous studies (Schwartz et al., 2012; Strus and Cieciuch, 2017; Czyżkowska and Cieciuch, 2020), to correct for individual differences in using the response scales, each participant’s responses were centered on their mean for all scales used in the present study. The mean of all items included in the scale was subtracted from each subscale score. For instance, the mean of all 57 value scores was subtracted from each higher-order value score.

### Data analysis

We tested connections among basic romantic motivations in the entire sample and separately for men and women. The analysis was conducted in two steps. First, we calculated the scores for each of the 14 basic romantic motivations as means of the corresponding items. Second, we tested the hypothesized circular structure of romantic motivations by applying multidimensional scaling (MDS) to the 14 basic romantic motivations. We used MDS because this analytical approach is useful for testing circumplex models. For such models, exploratory or confirmatory factor analyses are inappropriate because of the expected strong intercorrelations between variables (Schwartz et al., 2012; Cieciuch, 2017; Czyżkowska and Cieciuch, 2020). We used the Multidimensional Scale module in SPSS (Alscal Procedure Options) to conduct MDS.

We tested connections between values, romantic motivations, and mate preferences using Structural Equation Modeling in Mplus (Muthén and Muthén, 2012). Full information maximum likelihood estimation with robust standard errors was used to deal with missing data (Little and Rubin, 2019). The covariance structure of the model was evaluated using multiple fit indexes, and the following values were regarded as indicating a good fit: *χ2*/*df* < 3.0, *CFI* > 0.95, *TLI* > 0.95, and *RMSEA* < 0.05 (Geiser, 2012; Kelloway, 2014). The mediation effect of romantic motivations is corroborated when the indirect effect of values on mate preferences through romantic motivations is significant. Mediation is considered complete when the indirect effect of values on mate preferences is significant, and the direct effect of values on mate preferences is not significant. Mediation is considered partial when both the indirect and direct effects of values on mate preferences are significant. According to modern statistical literature, using SEM for testing mediation has numerous advantages over the method suggested by Baron and Kenny (Bollen and Pearl, 2012; Gunzler et al., 2013; Kelloway, 2014).

## Results

### The structure of romantic motivations

**Figure 1:**
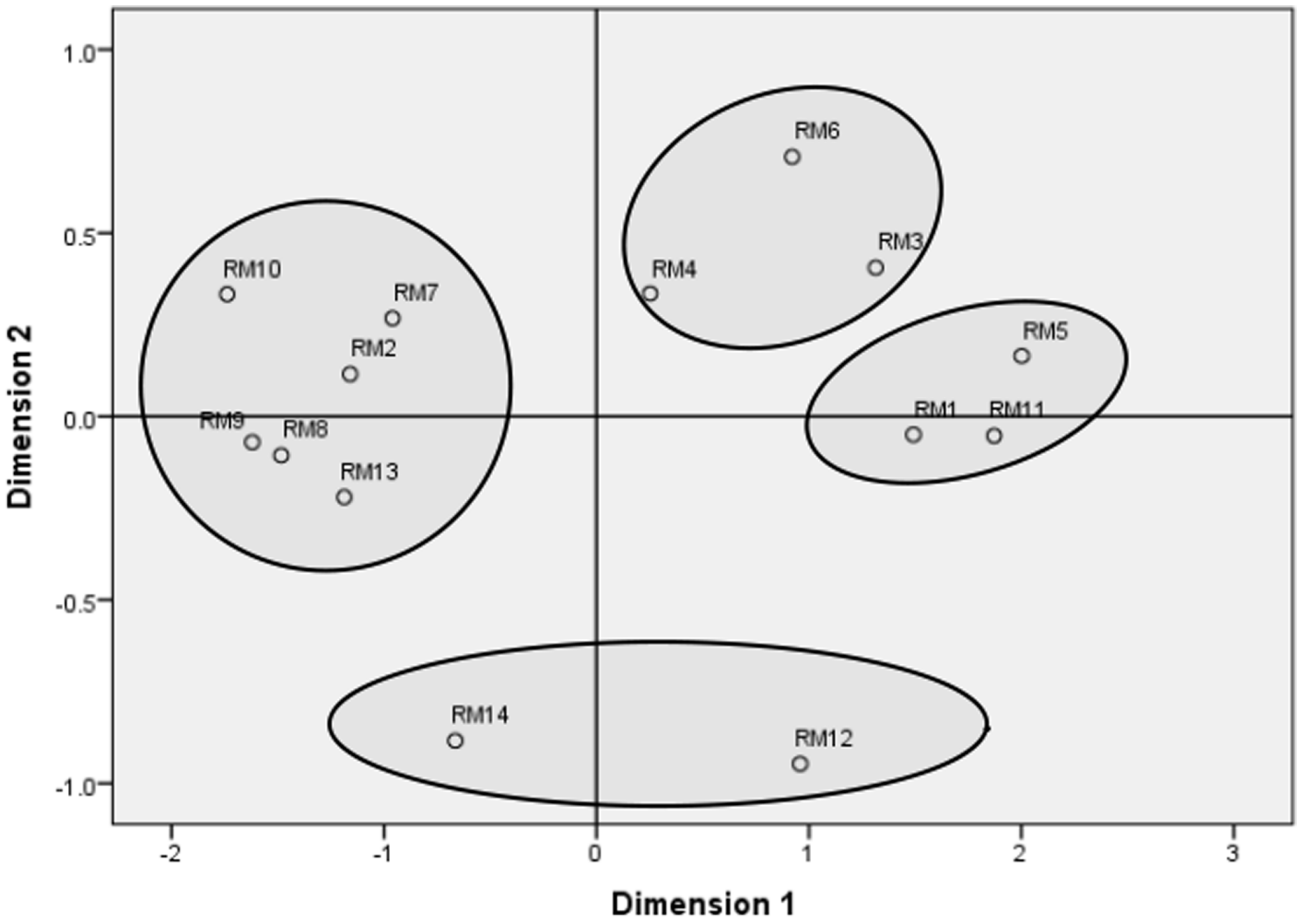
Multidimensional scaling configuration derived in two dimensions: the entire sample. RM 1, care for the other; RM 2, independence from parents; RM 3, psychological growth; RM 4, escape from loneliness; RM 5, feeling loved; RM 6, sexual satisfaction; RM 7, social advancement; RM 8, control over the other; RM 9, economic benefits; RM 10, respect; RM 11, emotional support; RM 12, childbearing and childrearing; RM 13, avoiding social pressure; RM 14, starting a family.

Figure 1 presents the MDS configuration for the entire sample. Appendix Figures A1, A2 present the MDS graphs separately for men and women. The configurations obtained separately for the two genders and the entire sample were similar. The MDS goodness of fit indexes demonstrated an excellent fit (the entire sample/men/women): Young’s stress = 0.024/0.023/0.026; Kruskal’s stress = 0.043/0.046/0.054.

The results confirmed our hypothesis that basic romantic motivations form a circumplex that may be partitioned into four clusters (higher-order romantic motivations): Love and Care, Sex and Adventure, Status and Resources, and Family and Children. The love and care cluster included three motivations: care for the other, feeling loved, and receiving emotional support. The sex and adventure cluster included three motivations: sexual satisfaction, escape from loneliness, and psychological growth. The status and resources cluster included six motivations: social advancement, control over the other, economic benefits, respect, independence from parents, and avoiding social pressure. Finally, the family and children cluster included two motivations: starting a family and finding a partner for childbearing and childrearing.

Comparing the obtained romantic motivations circumplex with our hypotheses, we found that 11 out of 14 motivations were in their hypothesized clusters, and no romantic motivation was in the cluster opposite the hypothesized. However, three basic motivations were not in the hypothesized but in an adjacent cluster: Independence from parents was in the status and resources, not the sex and adventure cluster; avoiding social pressure was in the status and resources, not the children and family cluster; and care for the other was in the love and care cluster, with two other basic romantic motivations of feeling loved and receiving emotional support. Therefore, the obtained results mainly corroborated the hypothesized structure of romantic motivations.

### Connections between romantic motivations and values

To test the connections between romantic motivations and values, we first calculated scores of four higher-order romantic motivations as means of the corresponding basic romantic motivations. After that, we calculated Pearson correlation coefficients between the four higher-order romantic motivations and four higher-order values. Table 2 presents the obtained results separately for men and women. For both genders (men/ women), Status and Resources romantic motivations were positively correlated with self-enhancement values (0.35/0.34) and negatively correlated with self-transcendence values (−0.23/–0.35). In addition, they were negatively correlated with openness to change values among men and women (−0.13/–0.09) and positively correlated with conservation values among women (0.12). For both genders, Love and Care motivations were positively correlated with self-transcendence (0.27/0.40) and openness to change values (0.14/0.08), and negatively correlated with self-enhancement (−0.33/–0.39) and conservation (−0.10/–0.13) values. For both genders, Family and Children motivations were positively correlated with conservation values (0.14/0.16). In addition, for men, they were positively correlated with self-transcendence (0.17) and negatively correlated with self-enhancement values (−0.19). Finally, for both genders, Sex and Adventure motivations were positively correlated with openness to change (0.21/0.21) and negatively correlated with conservation values (−0.12/–0.25). In addition, among women, these motivations were positively correlated with self-transcendence values (0.13). These findings corroborated our hypotheses regarding the pattern of connections between romantic motivations and values.

**Table 2:** Higher-order romantic motivations and values: Pearson correlation coefficients, means, and standard deviations.

Variables	SR	LC	FC	SA	O2CH	SENH	CONS	SETR	M(SD)
SR	1	−0.805^**^	0.167^**^	−0.303^**^	−0.093^*^	0.340^**^	0.119^**^	−0.351^**^	−1.30 (0.63)
LC	−0.834^**^	1	−0.216^**^	−0.009	0.078^*^	−0.386^**^	−0.127^**^	0.404^**^	1.08 (0.62)
FC	0.121^*^	−0.170^**^	1	−0.423^**^	−0.033	−0.023	0.158^**^	−0.070	−3.22 (1.31)
SA	−0.361^**^	0.068	−0.418^**^	1	0.205^**^	−0.022	−0.249^**^	0.129^**^	0.34 (0.62)
O2CH	−0.128^**^	0.144^**^	−0.091	0.207^**^	1	−0.027	−0.671^**^	0.072	0.25 (0.53)
SENH	0.354^**^	−0.331^**^	−0.186^**^	−0.063	0.062	1	−0.206^**^	−0.605^**^	−0.63 (0.69)
CONS	0.019	−0.100^*^	0.141^**^	−0.119^*^	−0.678^**^	−0.335^**^	1	−0.343^**^	−0.21 (0.50)
SETR	−0.231^**^	0.268^**^	0.170^**^	0.029	0.073	−0.618^**^	−0.274^**^	1	0.42 (0.42)
*M*(SD)	−1.29 (0.61)	1.26 (0.65)	−3.56 (1.28)	0.48 (0.61)	0.43 (0.55)	−0.61 (0.76)	−0.27 (0.53)	0.34 (0.47)	1

Women’s data are above the diagonal; men’s are below. SR, status and resources; LC, love and care; FC, family and children; SA, sex and adventure. O2CH, openness to change; SENH, self-enhancement; CONS, conservation; SETR, self-transcendence. *p < 0.05, *p < 0.01.

We conducted linear regressions to test the connections between values’ axes scores and romantic motivations while controlling for sociodemographic variables (Table 3). Status and resources romantic motivations were negatively associated with self-transcendence vs. self-enhancement (*β* = −0.30) and openness to change vs. conservation values (*β* = −0.09). Love and care motivations were positively associated with self-transcendence vs. self-enhancement (*β* = 0.35) and openness to change vs. conservation values (*β* = 0.09). Sex and adventure motivations were positively associated with openness to change vs. conservation values (*β* = 0.15). Finally, family and children motivations were positively associated with self-transcendence vs. self-enhancement values (*β* = 0.11). Values’ axes scores predicted romantic motivations over and above sociodemographic variables; the proportion of variance explained was 14–21%. The results corroborated our hypothesis that romantic motivations are associated with general motivational goals expressed in values when controlling for socio-demographic variables.

**Table 3:** Linear regressions: sociodemographic variables and value axes scores predicting higher-order romantic motivations.

Predicting variables	Status and resources		Love and care		Sex and adventure		Family and children	
	Stage I	Stage II	Stage I	Stage II	Stage I	Stage II	Stage I	Stage II
Sociodemographic variables								
Age	0.023	0.017	−0.049	−0.042	0.037	0.028	−0.007	−0.003
Gender (1-m, 2-f)	−0.036	−0.021	−0.106***	−0.124***	−0.077**	−0.062*	0.085**	0.077**
Education	0.008	0.003	−0.018	−0.012	0.038	0.037	0.040	0.042
Ethnicity (1-Jews, 2-Arabs)	0.321***	0.246***	−0.235***	−0.149***	−0.289***	−0.296***	0.090**	0.119***
Level of religiosity	0.022	−0.003	−0.102**	−0.077*	−0.158***	−0.104**	0.290***	0.290***
Value axes scores								
O2CH – CONS		−0.089**		0.091**		0.153***		0.006
SETR – SENH		−0.303***		0.347***		−0.009		0.113***
*R^2^*	0.10	0.20	0.09	0.21	0.15	0.17	0.13	0.14
*Adjusted R^2^*	0.10	0.19	0.09	0.21	0.15	0.17	0.12	0.13
*F*	*F*(5;1,055) = 24.6; *p* < 0.001	*F*(7;1,053) = 36.4; *p* < 0.001	*F*(5;1,055) = 21.8; *p* < 0.001	*F*(7;1,053) = 40.2; *p* < 0.001	*F*(5;1,055) = 37.2; *p* < 0.001	*F*(7;1,053) = 30.9; *p* < 0.001	*F*(5;1,055) = 30.0; *p* < 0.001	*F*(7;1,053) = 23.8; *p* < 0.001
*F_ΔR_^2^*		*F*(2;1,053) = 59.1; *p* < 0.001		*F*(2;1,053) = 78.2; *p* < 0.001		*F*(2;1,053) = 12.8; *p* < 0.001		*F*(2;1,053) = 7.32; *p* = 0.001

*p < 0.05; **p < 0.01; ***p < 0.001. O2CH – CONS, openness to change vs. conservation axis score; SETR – SENH, self-transcendence vs. self-enhancement axis score. The table presents standardized regression coefficients.

Considering the effect of socio-demographic variables on romantic motivations, we found that age and education were not related to romantic motivations. Compared to men, women reported higher importance of family and children (*β* = 0.08) and lower importance of love and care (*β* = −0.12) and sex and adventure (*β* = −0.06) motivations; no gender difference in status and resources motivations was found. Comparing Israeli Arabs and Jews, we found that Arabs reported higher importance of status and resources (*β* = 0.25) and family and children (*β* = 0.12) and lower importance of sex and adventure (*β* = −0.30) and love and care (*β* = −0.15) romantic motivations. Finally, the level of religiosity was positively associated with family and children (*β* = 0.29) and negatively associated with sex and adventure (*β* = −0.10) and love and care (*β* = −0.08) romantic motivations. Thus, our hypotheses regarding the effects of socio-demographic variables on romantic motivations were partly corroborated.^2^

### Connections with mate preferences

First, we calculated two romantic motivations axes scores – love and care vs. status and resources and sex and adventure vs. family and children – subtracting one pole score from the other. We further calculated Pearson correlation coefficients separately for men and women to test the connections between values and romantic motivations axes scores and mate preferences (Table 4). The pattern of connections was similar for the two genders, with several exceptions (men/women). The importance of the romantic partner’s status was correlated with love and care vs. status and resources (−0.31/–0.28), sex and adventure vs. family and children (−0.07, ns/–0.22), openness to change vs. conservation (−0.21/–0.27), and self-transcendence vs. self-enhancement (−0.26/–0.41). The importance of the romantic partner’s physical attractiveness was correlated with love and care vs. status and resources (0.09, ns/ 0.12), sex and adventure vs. family and children (0.30/0.22), openness to change vs. conservation (0.23/0.17), and self-transcendence vs. self-enhancement (−0.17/–0.01, ns). Finally, the importance of the mate’s similarity was correlated with love and care vs. status and resources (−0.30/–0.30), openness to change vs. conservation (−0.12/–0.16), and self-transcendence vs. self-enhancement (−0.17/–0.18). Thus, our hypotheses regarding the connections of mate preferences with values and romantic motivations were mostly corroborated.

**Table 4:** Mate preferences, romantic motivations, and values axes scores: Pearson correlation coefficients, means, and standard deviations.

Variables	LC – SR	SA – FC	O2CH – CONS	SETR – SENH	Status	Attractiveness	Similarity	*M*(SD)
LC – SR	1	0.215^***^	0.119^**^	0.429^***^	−0.277^****^	0.116^**^	−0.300^***^	1.19 (0.59)
SA – FC	0.202^***^	1	0.172^***^	0.036	−0.223^**^	0.215^***^	−0.067	3.57 (1.67)
O2CH – CONS	0.113^*^	0.166^***^	1	0.028	−0.266^***^	0.174^***^	−0.158^**^	0.45 (0.94)
SETR – SENH	0.354^***^	−0.135^***^	−0.067	1	−0.408^***^	−0.013	−0.179^***^	1.06 (0.99)
Status	−0.306^***^	−0.065	−0.206^***^	−0.257^***^	1	−0.052	−0.065	4.06 (1.08)
Attractiveness	0.086	0.301^***^	0.225^***^	−0.173^***^	−0.186^***^	1	−0.269^***^	4.63 (0.93)
Similarity	−0.303^***^	−0.018	−0.118^*^	−0.172^***^	−0.071	−0.167^***^	1	3.91 (1.06)
*M*(SD)	1.28 (0.61)	4.04 (1.64)	0.69 (0.99)	0.95 (1.11)	3.32 (1.22)	4.51 (1.10)	3.55 (1.16)	1

Women’s data are above the diagonal; men’s are below. O2CH – CONS, openness to change vs. conservation axis score; SETR – SENH, self-transcendence vs. self-enhancement axis score. LC – SR, love and care vs. status and resources axis score; SA – FC, sex and adventure vs. family and children axis score. *p < 0.05, *p < 0.01, *p < 0.001.

We conducted linear regressions to test the connections between romantic motivations and values axes scores and mate preferences while controlling for sociodemographic variables (Table 5). Status was connected to the openness to change vs. conservation (*β* = −0.19) and self-transcendence vs. self-enhancement value axes (*β* = −0.26). In addition, it was connected to love and care vs. status and resources romantic motivations (*β* = −0.11). Attractiveness was connected to the openness to change vs. conservation (*β* = 0.13) and self-transcendence vs. self-enhancement values axes (−0.13). In addition, it was connected to sex and adventure vs. family and children romantic motivations (*β* = 0.17). Finally, the similarity was connected to the openness to change vs. conservation values axis (*β* = −0.13) and love and care vs. status and resources romantic motivations (*β* = −0.24). Values predicted mate preferences over and above sociodemographic variables and romantic motivations predicted mate preferences over and above sociodemographic variables and values, corroborating our hypotheses.

**Table 5:** Linear regression analysis: sociodemographic variables, values, and romantic motivations axes scores predicting mate preferences.

	Status			Attractiveness			Similarity		
Predicting variables	Stage I	Stage II	Stage III	Stage I	Stage II	Stage III	Stage I	Stage II	Stage III
Sociodemographic variables								
Age	−0.035	−0.034	−0.037	0.160^***^	0.147^***^	0.147^***^	−0.060	−0.056	−0.064^*^
Gender (1-m, 2-f)	0.273^***^	0.276^***^	0.265^***^	−0.088^**^	−0.065^*^	−0.047	0.014	0.010	0.000
Education	−0.005	−0.010	−0.012	0.005	0.002	0.006	0.115^***^	0.113^***^	0.112^***^
Ethnicity (1-Jews, 2-Arabs)	0.199^***^	0.129^***^	0.093^**^	−0.126^***^	−0.117^***^	−0.163^***^	0.197^***^	0.168^***^	0.127^***^
Level of religiosity	0.124^***^	0.058^*^	0.035	−0.092^**^	−0.039	0.009	−0.039	−0.090^**^	−0.088^**^
Values								
O2CH – CONS		−0.206^***^	−0.192^***^		0.145^***^	0.131^***^		−0.153^***^	−0.132^***^
SETR – SENH		−0.295^***^	−0.264^***^		−0.130^***^	−0.134^***^		−0.131^***^	−0.046
Romantic motivations								
LC – SR			−0.110^***^			0.058			−0.237^***^
SA – FC			−0.068^*^			0.171^***^			0.041
*R^2^*	0.17	0.28	0.29	0.08	0.12	0.15	0.07	0.10	0.14
*Adjusted R^2^*	0.16	0.27	0.29	0.08	0.11	0.14	0.06	0.09	0.14
*F*	*F*(5;1,054) = 41.8; *p* < 0.001	*F*(7;1,052) = 58.1; *p* < 0.001	*F*(9;1,050) = 48.7; *p* < 0.001	*F*(5;1,054) = 19.3; *p* < 0.001	*F*(7;1,052) = 20.3; *p* < 0.001	*F*(9;1,050) = 20.4; *p* < 0.001	*F*(5;1,054) = 14.7; *p* < 0.001	*F*(7;1,052) = 16.6; *p* < 0.001	*F*(9;1,050) = 9.33; *p* < 0.001
*F_ΔR_^2^*		*F*(2;1,052) = 82.6; *p* < 0.001	*F*(2;1,050) = 11.5; *p* < 0.001		*F*(2;1,052) = 21.0; *p* < 0.001	*F*(2;1,050) = 18.4; *p* < 0.001		*F*(2;1,052) = 20.1; *p* < 0.001	*F*(2;1,050) = 21.0; *p* < 0.001

*p < 0.05; **p < 0.01; ***p < 0.001. O2CH – CONS, openness to change vs. conservation axis score; SETR – SENH, self-transcendence vs. self-enhancement axis score. LC – SR, love and care vs. status and resources axis score; SA – FC, sex and adventure vs. family and children axis score. The table presents standardized regression coefficients.

Considering the effect of socio-demographic variables on mate preferences, we found that age was significantly connected with the importance of attractiveness (*β* = 0.15) and similarity (*β* = −0.06) but not with status. Women ascribed higher importance to the status of their partners than men (*β* = 0.27), but there were no significant gender differences regarding other mate preferences. Education was positively connected with similarity (*β* = 0.11). Compared to Jews, Arabs ascribed higher importance to status (*β* = 0.09) and similarity (*β* = 0.13) and lower importance to attractiveness (*β* = −0.16). Taken alone, a higher level of religiosity was associated with higher importance of status (*β* = 0.12) and lower importance of attractiveness (*β* = −0.09); however, both effects of religiosity disappeared after including values and romantic motivations in the regression, and the connection with similarity became significant (*β* = −0.09).

### Direct and indirect effects of values on mate preferences

**Figure 2:**
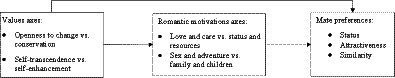
Path analysis: the hypothesized model.

**Figure 3:**
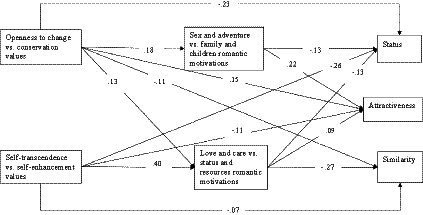
Path analysis: the trimmed model.

Figure 2 presents the hypothesized model that includes the following variables: two values axes (openness to change vs. conservation and self-transcendence vs. self-enhancement), two romantic motivations axes (love and care vs. status and resources and sex and adventure vs. family and children), and three mate characteristics (status, attractiveness, and similarity). After the model’s goodness-of-fit was established, aiming for the most parsimonious model, the initial research model was “trimmed,” i.e., all not significant paths were excluded (Kelloway, 2014). The trimmed model demonstrated an excellent fit: *χ^2^*(2) = 2.77, *p* = 0.250; *RMSEA* (*CI*) = 0.019 (0.000; 0.065); *CFI* = 0.999; *TLI* = 0.992. The proportion of variance explained was significant for all mate preferences: status (21%), attractiveness (11%), and similarity (11%). Figure 3 presents connections between variables in the trimmed model. As predicted, connections between romantic motivations and mate preferences were significant: Sex and adventure vs. family and children motivations were connected to status (*β* = −0.13) and attractiveness (*β* = 0.22); love and care vs. status and resources motivations were connected to status (*β* = −0.13), attractiveness (*β* = 0.09), and similarity (*β* = −0.27). In addition, direct connections between values and mate preferences were significant: Openness to change vs. conservation values were connected to status (*β* = −0.23), attractiveness (*β* = 0.15), and similarity (*β* = −0.11), and self-transcendence vs. self-enhancement values were directly connected to status (*β* = −0.26), attractiveness (*β* = −0.11), and similarity (*β* = −0.07). Finally, values were indirectly (through romantic motivations) connected to mate preferences: Openness to change vs. conservation values were indirectly connected to status (*β* = −0.040, *p* < 0.001), attractiveness (*β* = 0.050, *p* < 0.001), and similarity (*β* = −0.034, *p* < 0.001), and self-transcendence vs. self-enhancement values were indirectly connected to status (*β* = −0.050, *p* < 0.001), attractiveness (*β* = 0.034, *p* = 0.008), and similarity (*β* = −0.105, *p* < 0.001). These results corroborated the mediating hypothesis, indicating that romantic motivations partly mediate the connections between values and mate preferences.

## Discussion

### Structure of romantic motivations

In this study, we revealed a structure of romantic motivations. We found that 14 basic romantic motivations form four motivational clusters or higher-order motivations. The first cluster, *love and care*, includes three romantic motivations: caring for the other, feeling loved, and receiving emotional support. This motivational cluster expresses the desire to give and receive love and emotional support. The combination of giving and receiving motivations in one cluster is unusual because promoting one’s interests usually opposes caring for others (Schwartz, 2017). However, in romantic relationships, these motivations are complementary. Thus, romantic relationships differ from other interpersonal relationships in that they permit individuals simultaneously to care for each other and be cared for. We found that love and care is the most important motivation for seeking romantic relationships among young men and women. This finding indicates that the primary motivational goal of romantic relationships for both genders is giving and receiving love and emotional support, caring for the other, and being cared for. This finding corroborates the results of previous studies on the primacy of love in romantic relationships (Cohn, 2013).

The second cluster, *sex and adventure*, includes three romantic motivations: psychological growth, escape from loneliness, and sexual satisfaction. This romantic motivation reflects a desire to find a new experience, including a sexual one, that may lead to personal growth. The sex and adventure motivations are ranked second in importance among young men and women. The sex and adventure romantic motivations are compatible with love and care motivations, thus indicating that love and care usually accompany sex when people engage in romantic relationships (Townsend et al., 2020; Sorokowski et al., 2021; Thorpe and Kuperberg, 2021).

The third cluster *status and resources*, includes six romantic motivations: independence from parents, avoiding social pressure, social advancement, economic benefits, control over the other, and respect. These romantic motivations reflect the motivational goals of promoting one’s social status and obtaining resources through romantic relations. This cluster was ranked third in importance by both men and women. It strongly contradicted love and care romantic motivations among young men and women, thus indicating that these two motivations are incompatible. These findings indicate that an individual usually cannot give and receive love and care and simultaneously use a romantic partner to strengthen one’s social status and improve one’s economic conditions. These findings corroborate previous studies on social exchange theory that demonstrated the incompatibility of love and material resources exchange in interpersonal relationships (Mitchell et al., 2012).

The fourth cluster, *family and children*, includes motivations to raise a family and find a partner for childbearing and childrearing. This motivation is the least important among young men and women looking for a romantic partner. This finding is surprising, given the pronatalist character of the Israeli state (Waldman, 2006). However, it may be explained by the fact that family and children motivations relate to a distant future, whereas romantic relationships are mostly present-oriented. The family and children’s motivation strongly contradicted the sex and adventure motivation among both men and women, indicating that these motivations are incompatible in romantic relationships.

The system of compatibilities and conflicts between higher-order romantic motivations discovered in the present study indicates that romantic motivations exist in a two-dimensional space: one dimension running between love and care and status and resources poles, another – between sex and adventure and family and children’s poles. These dimensions are not orthogonal but rather slightly positively correlated. It means that people who are high on love and care motivations tend to be also high on sex and adventure motivations; thus, these motivations express compatible motivational goals. The same applies to status and resources and family and children motivations. This system of romantic motivations’ compatibilities and conflicts is similar among men and women. These findings are important because they demonstrate that romantic motivations constitute a meaningful system of congruencies and conflicts, not a unidimensional construct assumed in some previous studies (Eekelaar, 2007; Cohn, 2013; Park and Rosén, 2013; Carter, 2018). Thus, people cannot simultaneously achieve all possible motivational goals in romantic relationships and must trade between conflicting goals.

### Romantic motivations and values

In this study, we assumed that romantic motivations express general motivational goals in the context of romantic relationships. We corroborated this assumption by finding a meaningful pattern of connections between romantic motivations and personal value preferences. We found that love and care romantic motivations in both genders are associated with a preference for self-transcendence vs. self-enhancement values and, to a lesser degree, with a preference for openness to change vs. conservation values. Thus, love and care romantic motivations express the general motivational goals of caring for others and transcending one’s interests for the sake of others. In a more general sense, love and care motivations express general motivational goals of psychological growth and development and might be found more often in people with a relatively low level of anxiety (Schwartz et al., 2012).

Sex and adventure romantic motivations are associated with a high preference for openness to change vs. conservation values in both genders. Thus, sex and adventure romantic motivations express the general motivational goals of growth and self-actualization through looking for new experiences. In addition, among women but not men, sex and adventure motivations are associated with a high preference for self-transcendence values. This finding indicates that sex and adventure romantic motivations have different meanings for men and women, being more other-focused among women. These findings corroborate previous studies on gender differences in sexual relationships that demonstrated that women more often use sex to express caring for their partner (Petersen and Hyde, 2011). The present study provides a motivational explanation for the previous findings, indicating that sex for women may be a way of caring for others (Thompson and O’Sullivan, 2012).

Status and resources romantic motivations were associated with a high preference for self-enhancement vs. self-transcendence and a high preference for conservation vs. openness to change values for both genders. Therefore, these romantic motivations express anxiety avoidance and self-protection as general motivational goals in romantic relationships. They might be more important among people with a higher level of anxiety and a history of traumatization (in the family, previous romantic relationships, or in general). However, this hypothesis needs testing in further research.

Family and children’s romantic motivations were associated with a higher preference for conservation vs. openness to change values for both genders. However, it was also associated with a higher preference for self-transcendence vs. self-enhancement values for men. These findings shed light on the gender differences in romantic relationships. Men who aim to find a romantic partner to raise the family and children tend to be more ready to transcend their interests for the sake of others. However, no such tendency exists among women who seek romantic relationships to establish a family and raise children. These findings may reflect different social norms related to raising families and children. Men may perceive family and children as requiring them to give up some of their interests and care more for others, while women may perceive raising family and children as serving their own interests and the interests of the other (Buss et al., 2001; Hurt, 2014; Gittins, 2017).

### Values, romantic motivations, and mate preferences

Romantic motivations and values predicted mate preferences over and above sociodemographic variables. Specifically, the importance of the partner’s social status was associated with status and resources romantic motivations in both genders and with family and children’s romantic motivations among women. Thus, people whose goal in romantic relationships is to elevate their social status and resources are looking for a partner with high social status. The difference between men and women in the connection between the partner’s status and the family and children’s romantic motivation corroborates the previously found gender differences in the meaning of family and children for the two genders, with women preferring a more resourceful partner for raising a family and giving birth to children (Buss et al., 2001; Thompson and O’Sullivan, 2012). Finally, the importance of the social status of the romantic partner was associated with self-enhancement and conservation values among men and women. These findings indicate that romantic relationships with high-status partners enhance individuals’ social status and control over resources. In addition, more conservative people probably tend to choose a high-status partner because it matches social expectations (Walter et al., 2020; Cooperman and Waller, 2022).

The importance of physical attractiveness in the romantic partner was associated with sex and adventure romantic motivations among both genders and with love and care motivations among women. These findings indicate that men and women seeking sexual satisfaction prefer an attractive partner. However, it also indicates that women, but not men, who are seeking love in romantic relationships prefer an attractive partner. It indicates that women, but not men, find it easier to love and care for an attractive partner. These findings are interesting and require further investigation. The importance of the partner’s physical attractiveness was associated with openness to change values in both genders and self-enhancement values among men. These findings indicate that an attractive partner permits both genders to obtain new experiences more easily. However, our findings also demonstrate that an attractive partner is a status symbol for men but not women, as shown in some previous studies (Hurt, 2014; Gittins, 2017).

We expected that the preference for similarity in a romantic partner would be connected to romantic motivations associated with conservation values, i.e., family and children motivations. However, we found that the similarity in mate preference contradicts love and care motivations and is associated with status and resources romantic motivations in both genders. This finding indicates that individuals looking for love and care in romantic relationships may be happy with a partner different from themselves. However, those seeking romantic relationships to increase their status and resources feel more confident with a similar partner. The importance of similarity in the romantic partner is also associated with conservation and self-enhancement values in both genders. This is probably because having a partner similar to oneself is socially normative, preserves the existing social order and status quo, and is compatible with obtaining resources and dominating other people. These findings indicate that individuals are more confident with and feel less threatened by a partner similar to them, probably because self-enhancement and conservation values are associated with a high level of anxiety (Schwartz, 2017). The present study findings highlight the interpersonal differences in assortative mating and reveal their motivational roots. Thus, they advance the previous studies that focused on the universal aspects of assortative mating (Buss et al., 2001; Thompson and O’Sullivan, 2012; Luo, 2017; Cooperman and Waller, 2022).

Our study confirmed that romantic motivations partly mediate the connection between personal value preferences and the sought-after partner’s characteristics. This finding indicates that in romantic relationships, people seek a partner whose characteristics help them attain general motivational goals expressed in value preferences and specific motivational goals relevant to romantic relationships. This finding is important because it not only reveals the context-specific mechanism related to romantic relationships but also advances our understanding of the valence mechanism in general (Hitlin, 2003; Sagiv and Roccas, 2021), demonstrating that general motivational goals may affect attitudes and behavior indirectly through their effect on context-specific motivational goals.

### The effect of sociodemographic variables on romantic motivations and mate preferences

We found significant effects of several socio-demographic variables on romantic motivations. Love and care and sex and adventure motivations were more important to men, while family and children motivations were more important to women. The higher importance of sex for men and long-term relationships for women is a long-established finding (James, 2010; Carter, 2018). However, the higher importance of love and care motivations for men in the present study is surprising. Our findings may reflect a culturally specific phenomenon (Lavee and Katz, 2003; Bystrov, 2012) or indicate the changes in modern youth (Gittins, 2017). In any case, this phenomenon requires further research.

We found that ethnicity is an important factor related to romantic motivations. Comparing the two ethnic groups, Arab Israelis reported higher importance of status and resources and family and children motivations, while Jews reported higher importance of love and care and sex and adventure motivations. Previous studies show that, compared to Israeli Jewish culture, Arab Israeli culture is characterized by higher preferences for conservation and self-enhancement values (Sagiv & Schwartz, 1995; Sagy et al., 2001). Similar results have been obtained in our study. Thus, the differences in romantic motivations reflect differences in values between the two main ethnic groups in Israel. However, it is important to note that the ranking of romantic motivations was similar among Jewish and Arab Israelis, which may indicate the existence of universal aspects of romantic motivations. Further cross-cultural studies are required to test the external validity of our findings.

A higher religiosity level was associated with higher importance of family and children motivations and lower importance of sex and adventure and love and care motivations. The strong positive connection between conservative values and religiosity may explain these findings (Schwartz, 2017). Neither age nor education was related to romantic motivations. However, it is possible that we could not detect the connections with these variables because our sample was restricted in both age (18–30) and education (3/4 post-secondary education).

We found several significant connections between socio-demographic variables and mate preferences. The partner’s physical attractiveness is more important for older people. Status is more important to women as compared to men. Similarity is more important to more educated people. Compared to Jews, status and similarity are more important to Arabs, while the partner’s physical attractiveness is more important to Jews. These findings corroborate previous ones on mate preferences in individualistic vs. collectivistic cultural groups, and they may be explained by social norms and values existing in each culture (Reneflot, 2006; Gassanov et al., 2008; Carter, 2018).

Men’s and women’s mate preferences have several similarities and differences. Physical attractiveness is the most important characteristic of the partner for both men and women. However, similarity is the second most desirable characteristic for men, while the partner’s status is the second most important characteristic for women. The rank differences in mate preferences between men and women were identical among Jewish and Arab Israelis. Our findings regarding gender differences in the importance of physical attractiveness for men and women in the present study differ from previous studies that found that the partner’s physical attractiveness was more important for men than women (Buss et al., 2001). However, our findings corroborate the results of studies on this issue that used the Implicit Association Test (Thompson and O’Sullivan, 2012). Our findings may be culture-specific or indicate changes in the present generation (Twenge, 2013). Further cross-cultural studies of this issue are required.

### Limitations and suggestions for further research

Several limitations of the study must be considered. First, it was correlational; therefore, causal inferences cannot be drawn from the results. Future longitudinal research would represent a significant advancement in the current findings. The second limitation of the present study is its sample, which was large but not random. The lack of control over the sample may raise generalizability issues. Further research should be based on representative samples. The third limitation relates to the research population. The suggested theoretical model was tested only in one country – Israel. Testing it in other countries would be essential to its generalization. The fourth limitation of the present study is that we focused on individual-level factors and did not investigate the macro and mezzo-level factors that might affect romantic motivations and mate preferences. Finally, the present study focused on young people with no girl/boyfriend. Further studies may investigate changes in motivations and mate preferences in different stages of romantic relationships: before their beginning, with a boy/girlfriend, during cohabitation, and after marriage.

## Conclusion

In this study, we investigated the motivational aspects of romantic relationships. We conceptualized romantic motivations as context-specific motivations derived from general motivational goals reflected in personal value preferences. We revealed a system of affinities and conflicts between romantic motivations and confirmed the existence of four clusters of romantic motivations: love and care, family and children, status and resources, and sex and adventure. We demonstrated that romantic motivational clusters form a meaningful pattern of connections with higher-order values. Thus, we could assemble many romantic motivations into a limited number of higher-order motivations and relate them to general motivational goals expressed in values. Finally, we demonstrated that values and romantic motivations predict mate preferences – the sought-after characteristics of the romantic partner. The results obtained in the present study allow us to understand interpersonal differences in romantic motivations and mate preferences. The study’s findings advance the values theory and our understanding of the valence principle, unveiling the connections between general motivations, context-specific motivations, and context-specific attitudes and behavior. Thus, our findings provide a solid basis for further research on general and context-specific motivations in interpersonal relationships. A better understanding of romantic motivations and their connections with mate preferences will be helpful in youth counseling to promote satisfactory decisions regarding dating and ongoing relationships. It will also allow helping professionals to develop interventions facilitating the psychological adjustment of young people in the context of romantic relationships.

## Data availability statement

The raw data supporting the conclusions of this article will be made available by the authors, without undue reservation.

## Ethics statement

The studies involving humans were approved by Tel Aviv University institutional review board. The studies were conducted in accordance with the local legislation and institutional requirements. The participants provided their written informed consent to participate in this study.

## Author contributions

ET: Conceptualization, Data curation, Formal analysis, Funding acquisition, Investigation, Methodology, Project administration, Resources, Writing – original draft.

## Footnotes

^2^We found significant differences in value preferences between Israeli Jews and Arabs. Jews reported higher preferences for openness to change [*M(SD)*_J_ = 0.35(0.58) vs. *M(SD)*_A_ = 0.25(0.45), *t*(1106) = 2.94, *p* = 0.003] and self-transcendence values [*M(SD)*_J_ = 0.45(0.46) vs. *M(SD)*_A_ = 0.25(0.36), *t*(1108) = 7.92, *p* < 0.001]. In addition, Jews reported lower preferences for conservation [*M(SD)*_J_ = −0.25(0.55) vs. *M(SD)*_A_ = −0.17(0.41), *t*(1104) = −2.80, *p* = 0.005] and self-enhancement values [*M(SD)*_J_ = −0.73(0.46) vs. *M(SD)*_A_ = −0.39(0.36), *t*(1104) = −7.98, *p* < 0.001].
