# Supplementary material for: The psychology of romantic relationships: motivations and mate preferences
Source: Front Psychol. 2023 Nov 28;14:1273607. doi: 10.3389/fpsyg.2023.1273607 (PMC10713733; doi:10.3389/fpsyg.2023.1273607)
Supplement: Supplementary file 1 [file Data_Sheet_1.docx]

**Appendix**

*Table A1. Basic Romantic Motivations and Scale Items*

Young men/ women have different reasons for wanting to find a girl/boyfriend. Why do you want to find a girl/boyfriend? To what extent are each of the items listed below important to you in searching for a girl/boyfriend? (1 – *not important at all* to 6 – *very important*)

| **Basic romantic motivations (Cronbach’s α men/ women)** | **Scale items** |
| --- | --- |
| **Psychological growth (.92/ .90)** | 1. To promote my self-development. |
|  | 1. To learn more about life. |
|  | 1. To obtain new life experience. |
|  | 1. To develop my personality. |
|  | 1. To achieve self-fulfillment. |
| **Independence from parents (.90/ .89)** | 1. To obtain psychological independence from my parents. |
|  | 1. To separate physically from my parents. |
|  | 1. To obtain financial independence from my parents. |
|  | 1. To free me from parental control. |
| **Escape from loneliness (.84/ .86)** | 1. To avoid boredom. |
|  | 1. To not be alone. |
|  | 1. To escape from loneliness. |
|  | 1. To have a companion who will entertain me. |
|  | 1. To have somebody to spend time with. |
|  | 1. To have somebody to talk with. |
| **Sexual satisfaction (.92/ .92)** | 1. To fulfill my sexual needs. |
|  | 1. To have a reliable sexual partner. |
|  | 1. To have good sex. |
|  | 1. To normalize my sexual life. |
|  | 1. To have sex without social condemnation. |
|  | 1. To have sex whenever I want. |
| **Social advancement (.88/ .86)** | 1. To raise my social status through relationships. |
|  | 1. To have somebody to help me with my work or/and study. |
|  | 1. To boost my career. |
|  | 1. To raise my sense of competence. |
|  | 1. To raise my sense of self-worth. |
| **Control over the other (.93/ .91)** | 1. To have somebody who will do everything I tell him to do. |
|  | 1. To have somebody who belongs only to me. |
|  | 1. To ensure my exclusive rights to somebody. |
|  | 1. To have somebody over whom I have control. |
|  | 1. To have somebody who will fulfill my desires. |
| **Economic benefits (.93/ .93)** | 1. To have somebody who will buy me things and pay my bills. |
|  | 1. To have somebody whose resources I may use for myself. |
|  | 1. To have somebody to help me with household chores (e.g., cleaning and cooking). |
|  | 1. To improve my economic conditions. |
|  | 1. To increase my financial security. |
|  | 1. To receive financial support. |
|  | 1. To obtain a place to live. |
|  | 1. To save on the cost of living. |
| **Respect (.91/ .90)** | 1. To be respected by others for my success in finding a partner. |
|  | 1. To make others envy me for having a partner. |
|  | 1. To raise my social status by finding a partner. |
| **Emotional support (.94/ .95)** | 1. To have somebody I can trust. |
|  | 1. To feel accepted despite my shortcomings. |
|  | 1. To get emotional support. |
|  | 1. To have somebody who is always by my side. |
|  | 1. To have somebody who cares for me. |
|  | 1. To have a sympathetic ear. |
|  | 1. To ensure a sense of security. |
|  | 1. To ensure a sense of stability. |
|  | 1. To find somebody who will take care of my needs. |
| **Feeling loved (.92/ .91)** | 1. To find somebody who will make me feel the joy of life. |
|  | 1. To find somebody who will make me feel happy. |
|  | 1. To feel loved. |
|  | 1. To feel admired. |
|  | 1. To feel accepted. |
|  | 1. To feel peaceful. |
| **Starting a family (.91/ .88)** | 1. To fulfill religious commandments of finding a husband/wife. |
|  | 1. To follow the tradition of having a partner who will become a husband/wife. |
|  | 1. To follow the religious commandment of starting a family. |
| **Childbearing and childrearing (.85/ .91)** | 1. To have somebody with whom I can bear children in wedlock. |
|  | 1. To have somebody to support my future children financially. |
|  | 1. To have somebody to raise children with. |
|  | 1. To ensure that my future children will have two parents. |
|  | 1. To ensure financial security for my future children. |
| **Avoiding social pressure (.87/ .88)** | 1. To satisfy my parents’ expectations of me having a partner. |
|  | 1. To follow the societal norm of having a partner. |
|  | 1. To fulfill my sense of duty by starting a family. |
|  | 1. To fulfill my friends’ expectations of me having a partner. |
| **Care for the other (.89/ .87)** | 1. To have somebody to care for. |
|  | 1. To have somebody to respect. |
|  | 1. To have somebody to love. |
|  | 1. To have somebody to adore. |
|  | 1. To make somebody happy. |
|  | 1. To make somebody feel desirable. |

Table A2. *Mate Preference Scale: Exploratory Factor Analysis (men/ women)*

How important is it to you that your boy/girlfriend has the following characteristics? ‎(1 – *not important at all* to 6 – *very important*)‎

| **Items** | **Factor 1: Status** | **Factor 2: Attractiveness** | **Factor 3: Similarity** |
| --- | --- | --- | --- |
| Earns well | .873/ ‎.862‎ | ‎‎ |  |
| Have a good career ahead | .891/ ‎.851‎ |  |  |
| Has a high social status | .651/ ‎.754‎ |  |  |
| From a good family | .583/ .713 |  |  |
| Looks good |  | .905/ ‎.739‎ |  |
| Healthy |  | .727/ ‎.595‎ |  |
| Sexy |  | .772/ ‎.833‎ |  |
| Has a personality similar to yours |  |  | .593/ ‎.701‎ |
| Has political views similar to yours |  |  | .747/ ‎.760‎ |
| Is similar to you religiously |  |  | .847/ ‎.733‎ |
| Has education similar to yours |  |  | .676/ ‎.577‎ |
| Has interests similar to yours |  |  | .699/ ‎.657‎ |
| *Eigenvalue* | 5.16/ 4.70 | 1.36/ 1.54 | 1.18/ 1.06 |
| *Cumulative variance explained, %* | 43%/ 39% | 54%/ 52% | 64%/ 61% |

*Note*: Scale items are back-translated from Hebrew. Extraction method: a principal component analysis. Rotation method: Oblimin with Kaiser normalization. Loadings smaller than 0.30 are suppressed.

Figure A1. *Multidimensional Scaling Configuration Derived in Two Dimensions: Men*


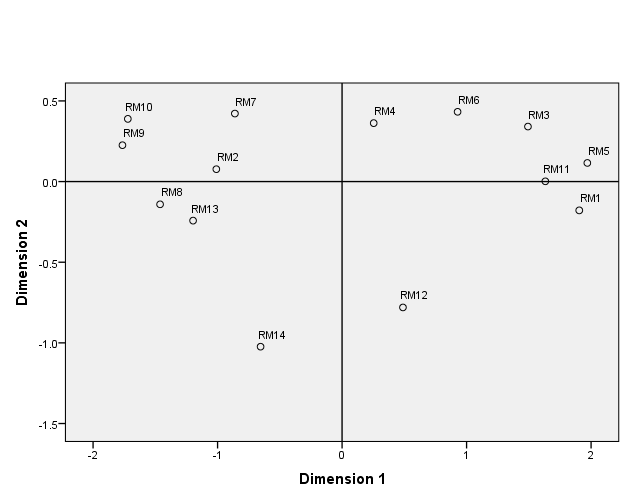


*Note*: RM 1 – Care for the other, RM 2 – Independence from parents, RM 3 – Psychological growth, RM 4 – Escape from loneliness, RM 5 – Feeling loved, RM 6 – Sexual satisfaction, RM 7 – Social advancement, RM 8 – Control over the other, RM 9 – Economic benefits, RM 10 – Respect, RM 11 – Emotional support, RM 12 – Childbearing and childrearing, RM 13 – Avoiding social pressure, RM 14 – Starting a family.

Figure A2. *Multidimensional Scaling Configuration Derived in Two Dimensions: Women*


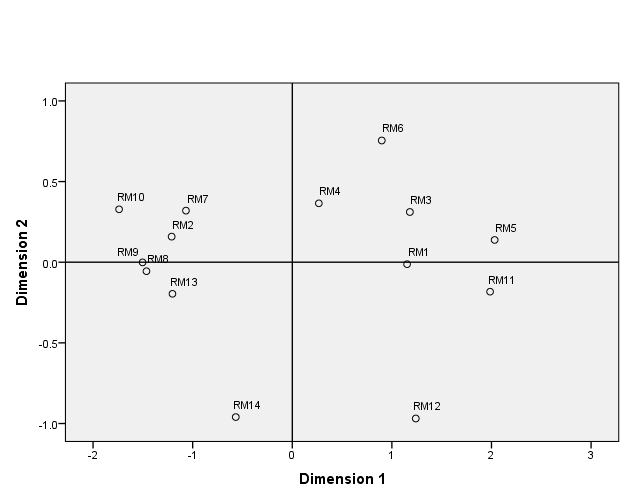


*Note*: RM 1 – Care for the other, RM 2 – Independence from parents, RM 3 – Psychological growth, RM 4 – Escape from loneliness, RM 5 – Feeling loved, RM 6 – Sexual satisfaction, RM 7 – Social advancement, RM 8 – Control over the other, RM 9 – Economic benefits, RM 10 – Respect, RM 11 – Emotional support, RM 12 – Childbearing and childrearing, RM 13 – Avoiding social pressure, RM 14 – Starting a family.
